# P-610. HPV vaccine knowledge, attitudes and practices among healthcare providers before introduction to the national schedule, Almaty, Kazakhstan - 2023

**DOI:** 10.1093/ofid/ofae631.808

**Published:** 2025-01-29

**Authors:** Feruza Ablimitova, Dilyara Nabirova, Roberta Horth, Ulyana Kirpicheva, Saya Gazezova, Manar Smagul

**Affiliations:** Central Asia FETP, Almaty, Almaty, Kazakhstan; CDC Central Asia office, Almaty, Almaty, Kazakhstan; US Centers for Disease Control and Prevention, Dulles, Virginia; ​Scientific and Practical Centre for Sanitary-Epidemiological Expertise and Monitoring, Almaty, Almaty, Kazakhstan; Central Asia Field Epidemiology Training Program, Almaty, Almaty, Kazakhstan; Scientific and practical center of sanitary-epidemiological examination and monitoring, branch of the National Center for Public Health, Almaty, Kazakhstan, Almaty, Almaty, Kazakhstan

## Abstract

**Background:**

Healthcare providers play a vital role in the success of new vaccine uptake. In 2024, Kazakhstan will introduce the human papillomavirus (HPV) vaccine into the immunization schedule after a 2013 pilot was unsuccessful due largely to widespread misinformation. We aimed to establish baseline knowledge, attitudes, and practices (KAP) related to HPV vaccination among providers to prepare for vaccine rollout.

**Figure:**
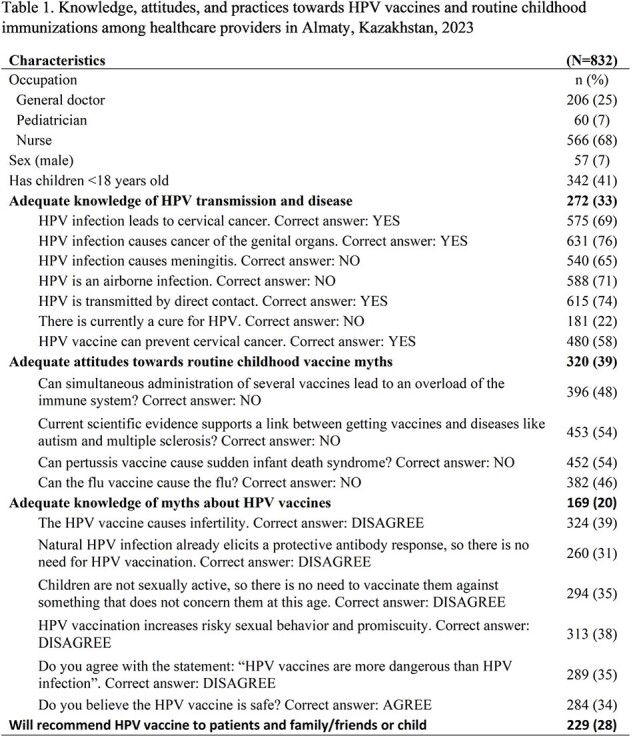

**Methods:**

We conducted a cross-sectional study in April-May 2023 of healthcare providers responsible for vaccinations in all 34 polyclinics that conduct vaccinations in Almaty. Providers completed anonymous questionnaires in group sessions. KAP domain scores >70% were considered adequate. We used multinomial logistic regression to assess factors associated with intent to recommend HPV vaccines; we presented odds ratios and 95% confidence intervals (95%CI).

**Figure:**
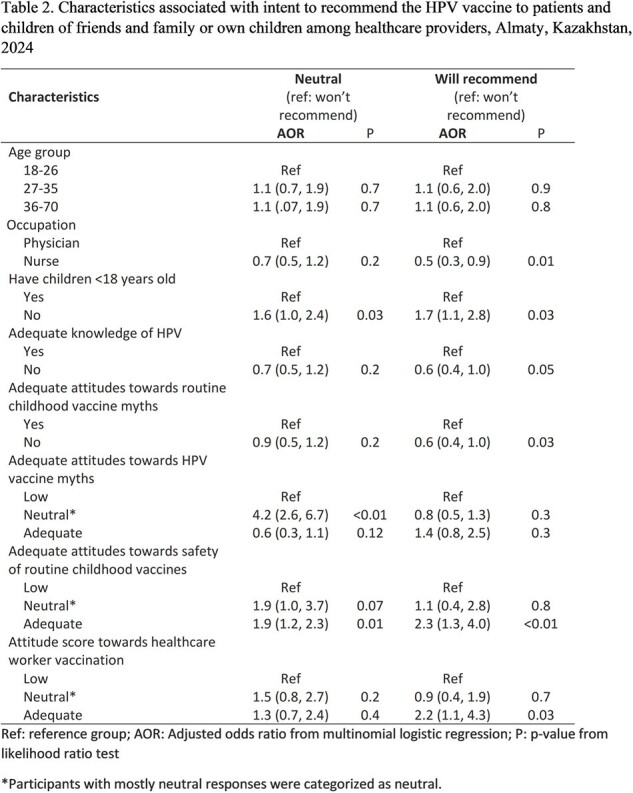

**Results:**

Of 832 participants, 68% were nurses and 41% had at least one < 18-year-old child (Table 1). One-third (33%) had adequate knowledge about HPV; specifically, 71% correctly believed HPV is not airborne, and 22% knew HPV infection is not currently curable. One-fifth (20%) had adequate attitudes towards HPV; and 39% knew that HPV vaccines don’t cause infertility. On questions related to routine childhood immunizations, 61% could not correctly dispel common myths. One-quarter (28%) would recommend the HPV vaccine to patients and to their own or friends’ children. The odds of recommending the HPV vaccine were lower for nurses compared to physicians (OR=0.5, 95%CI=0.3, 0.9), for providers with inadequate HPV knowledge compared to adequate (0.6, 95%CI=0.4, 1.0), and for those who believed common myths compared to those who didn’t (0.6, 95%CI=0.4, 1.0) (Table 2). The odds of recommending the vaccine were higher for providers with adequate attitudes towards HPV vaccine myths compared to those without (1.4, 95%CI=0.8, 2.5) and for those with adequate knowledge of childhood vaccine safety compared to those without (2.3, 95%CI= 1.3, 4.0).

**Conclusion:**

We found suboptimal KAP towards HPV vaccines. Results demonstrate the need to prioritize the rollout of a vaccine education, communication, and advocacy strategy to healthcare providers before the national HPV vaccine rollout.

**Disclosures:**

**Ulyana Kirpicheva, M.Sc. in FIeld Epidemiology**, Abbott Laboratories: Grant/Research Support

